# Recent Progress in the Development of Small-Molecule FtsZ Inhibitors as Chemical Tools for the Development of Novel Antibiotics

**DOI:** 10.3390/antibiotics8040217

**Published:** 2019-11-11

**Authors:** Laura Carro

**Affiliations:** 1 Laboratorio de Química Orgánica y Farmacéutica, Departamento de Ciencias Farmacéuticas, Facultad de Farmacia, Universidad de Salamanca, Campus Miguel de Unamuno, 37007 Salamanca, Spain; laura.carro.santos@usal.es; 2 School of Pharmacy, University of East Anglia, Norwich Research Park, Norwich NR4 7TJ, UK

**Keywords:** FtsZ, bacterial cell division, small-molecule inhibitors, new antibiotics, antibiotic resistance

## Abstract

Antibiotics are potent pharmacological weapons against bacterial pathogens, nevertheless their efficacy is becoming compromised due to the worldwide emergence and spread of multidrug-resistant bacteria or “superbugs”. Antibiotic resistance is rising to such dangerous levels that the treatment of bacterial infections is becoming a clinical challenge. Therefore, urgent action is needed to develop new generations of antibiotics that will help tackle this increasing and serious public health problem. Due to its essential role in bacterial cell division, the tubulin-like protein FtsZ has emerged as a promising target for the development of novel antibiotics with new mechanisms of action. This review highlights the medicinal chemistry efforts towards the identification of small-molecule FtsZ inhibitors with antibacterial activity in the last three years.

## 1. Introduction

The discovery of antibiotics unquestionably represented one of the most remarkable scientific breakthroughs of the twentieth century. Not only have they saved innumerable lives, but also they have permitted modern medicine procedures such as routine surgical operations, organ transplantations, or chemotherapy, among others. Lamentably, after many years of success in the fight against infectious diseases, the ever-increasing worldwide emergence and spread of “superbugs” (bacteria resistant to most of the antibiotics in current clinical use) is compromising our ability to treat infections. The impact that this situation has on the older population or immunocompromised patients is particularly problematic since treatment options are not available in certain cases [1].

Antibiotic resistance is inexorably rising to such threatening levels that the United Nations has estimated that it could kill ten million people by 2050. By this time, mortality due to antibiotic resistance is predicted to exceed that of cancer [2].

In February 2017, the World Health Organization (WHO) published a list of priority pathogens for which there is an urgent need for innovative treatments, and in which antibiotic-resistant bacteria were classified in three different categories: critical, high, and medium priority [3]. The WHO highlighted that the situation is highly critical for the clinically relevant multi-drug resistant ESKAPE pathogens: *Enterococcus faecium, Staphylococcus aureus, Klebsiella pneumoniae, Acinetobacter baumannii*, *Pseudomonas aeruginosa* and various *Enterobacteriaceae* species. This list is an excellent tool to incentivize antibacterial R&D and to raise awareness regarding the rational use of current and future antibacterial therapies in both humans and animals.

**Figure 1:**
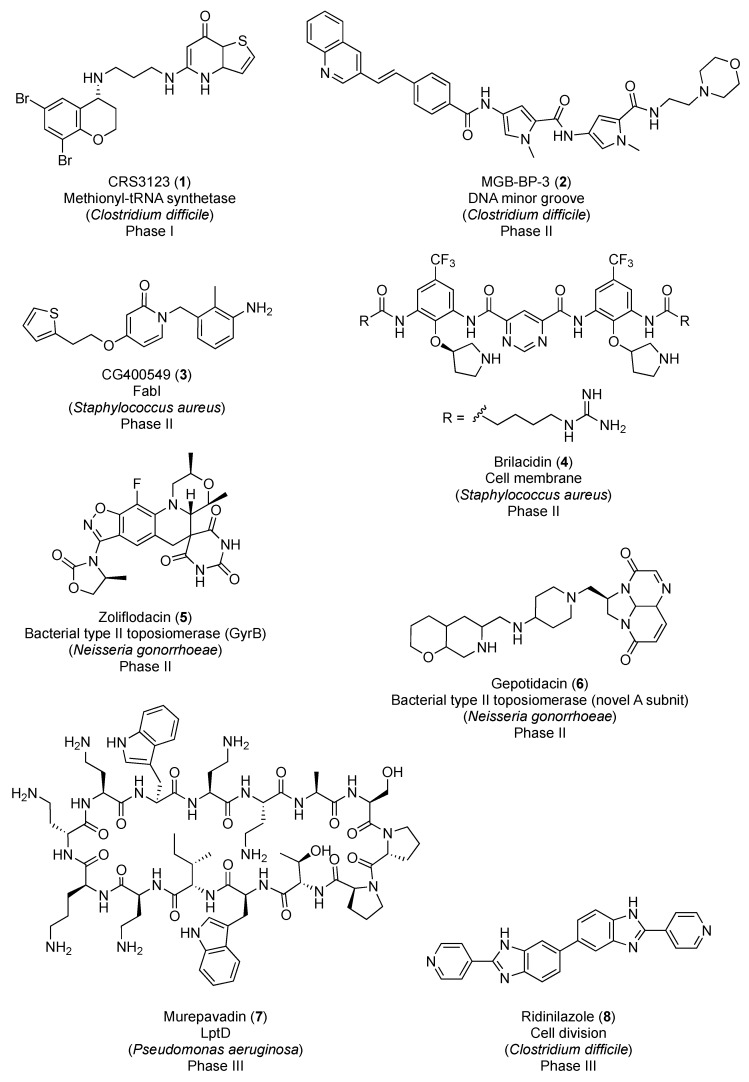
New-generation antibiotics in clinical trials.

In March 2019, the PEW Charitable Trusts published a resource with the antibiotics in clinical development as of December 2018 [4]. Among the forty-five antibacterial candidates, only eight operate via a completely novel mechanism of action. Figure 1 displays the chemical structures of these new-generation antibacterial candidates. CRS3123 (**1**), a methionyl-tRNA synthetase inhibitor developed by Crestone Inc., is currently in phase I for the treatment of *Clostridium difficile* infections [5]. In phase II of clinical development, there are five novel-mechanism antibiotics: MGB-BP-3 (**2**), CG400549 (**3**), brilacidin (**4**), zoliflodacin (**5**), and gepotidacin (**6**). MGB-BP-3 (**2**) from MGB Biopharma Ltd. targets the DNA minor groove and is likewise indicated in *C. difficile*-associated diarrhea [6]. The benzyl pyridinone CG400549 (**3**) is an inhibitor of the bacterial enoyl-ACP reductase (FabI) being developed by CrystalGenomics Inc. for the treatment of major cutaneous abscesses due to methicillin-resistant *Staphylococcus aureus* (MRSA) [7]. Brilacidin (**4**) is a defensin mimetic that is being investigated for acute bacterial skin and skin structure infections [8]. Zoliflodacin (**5**), a spiropyrimidinetrione from Entasis Therapeutics, targets the bacterial type II topoisomerase GyrB and shows promise in the treatment of uncomplicated gonorrhea [9]. Gepotidacin (**6**) is a first-in-class triazaacenaphthylene novel bacterial topoisomerase inhibitor (NBTI) from GSK that has recently completed phase II for the treatment of uncomplicated urogenital gonorrhea as well [10]. Murepavadin (**7**), an antimicrobial peptidomimetic in phase III for the treatment of pneumonia caused by *P. aeruginosa*, targets the β-barrel lipopolysaccharide-assembly protein D (LptD) [11]. And, last but not least, ridinilazole (**8**) is a bis-benzimidazole from Summit Therapeutics that inhibits cell division and, similar to candidates **1** and **2**, is currently being investigated for the treatment of *C. difficile* infections [12].

Considering that (a) the success rate for antibiotic development estimates that only one in five anti-infectives that enter clinical development will be approved for clinical use, and (b) these new antibacterials will add very little to the antibiotic armamentarium, the current antibacterial pipeline is undoubtedly insufficient to address the escalating threat of antimicrobial resistance [13,14]. Consequently, urgent action must be taken in order to develop new generations of antimicrobial chemical entities with innovative mechanisms of action that will help revitalize the dwindling antibacterial pipeline [15]. An example of these novel unconventional targets is FtsZ (filamenting temperature-sensitive protein Z), which, due to its crucial role in the bacterial cell division, has garnered increasing attention as a promising and exciting point of intervention to develop novel antibiotics [16,17,18,19]. 

In the hope of encouraging drug discovery in this field, this Review discusses the medicinal chemistry campaigns that led to the identification of small-molecule inhibitors of FtsZ in the last three years. Although the primary focus of this Review is examples of recently developed inhibitors of FtsZ, it also includes noteworthy examples identified in previous years. The structural characteristics, the discovery strategy, the characterization assay, the biological evaluation, and, if applicable, the structure-activity relationships (SAR) for each of the chemotypes are examined.

## 2. Review

### 2.1. Bacterial Cell Division and FtsZ as an Antibacterial Target

Prokaryotic cell division is orchestrated by the coordinated action of several proteins, termed the divisome. Among these, FtsZ is known to play an indispensable role in cell division in both Gram-positive and Gram-negative bacteria. Early in the division process, FtsZ self-assembles into a dynamic ring structure, the Z-ring, which operates as a focal point for the recruitment of other auxiliary division proteins. The divisome complex is responsible for the invagination and constriction of the cell membrane, before ultimately leading to bacterial cell division [20].

FtsZ is a GTP-ase structurally related to the eukaryotic protein tubulin [21,22]. Like tubulin, FtsZ polymerizes into protofilaments upon binding to GTP [23,24]; however, their cellular functions are different, and they share less than 20% sequence identity [25].

**Figure 2:**
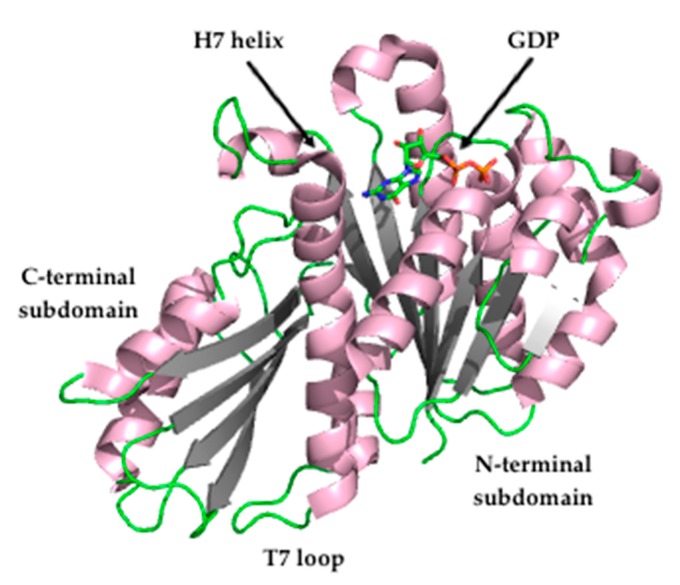
Crystal structure of *Staphylococcus aureus* FtsZ (PDB entry 3VO8) [27].

The crystal structure of FtsZ from *S. aureus* (*Sa*FtsZ) at 2.25 Å resolution (Figure 2) revealed that FtsZ is formed by two subdomains connected by a long core helix (H7 helix). The N-terminal portion comprises a six-stranded parallel β-sheet surrounded by two and three helices, and it contains the GTPase domain. On the other hand, the C-terminal domain is a four-stranded central β-sheet supported by two helices on one of the sides [26,27].

Recent studies have shown that FtsZ filaments display treadmilling behavior, in which FtsZ subunits are added to one of the filaments’ ends (plus end) while they are removed from the opposite filament’s end (minus end). This behavior is dependent on GTP activity and it has been found that it controls the peptidoglycan synthesis (the main component of the bacterial wall), and it limits the rate of bacterial cell division in *Bacillus subtilis* [28], but not in *Escherichia coli* [29]. In addition, it has recently been shown that *S. aureus* FtsZ adopts two conformations, open (found in FtsZ filaments) and closed (observed in monomeric FtsZ), and that this treadmilling is possible due to the fact that FtsZ undergoes the aforesaid conformational switch [30].

To date, reported FtsZ inhibitors have been documented to bind to: (i) the nucleotide-binding pocket in the N-terminal subdomain, such as the polyphenolic derivatives UCM05 and UCM44 [31], the marine natural product chrysophaentin A, and the synthetic hemi-chrysophaentin [32]; (ii) the cleft localized between the H7 helix and the C-terminal subdomain, such as the benzamide PC190723 [27,33]; (iii) the C-terminal subdomain, such as the peptide MciZ (mother cell inhibitor of FtsZ) [34]. Additionally, due to the role of FtsZ treadmilling behavior in the control of bacterial cell division, its inhibition may also be exploited for the development of antibacterial agents that impair bacterial cytokinesis.

Targeting FtsZ for the development of antibacterial leads offers several advantages. Firstly, it is essential for the multiplication and the viability of almost all prokaryotes. Secondly, it is highly conserved among bacteria. Thirdly, it differs sufficiently from its eukaryotic homolog tubulin, which offers the opportunity to develop FtsZ-targeting agents with limited toxicity to eukaryotic cells [35].

Taken together, it is not surprising that the development of FtsZ inhibitors has attracted a great deal of effort from several research groups. Nonetheless, for the purposes of this Review, the focus will be placed on the recent progress towards the development of small-molecule FtsZ inhibitors. Ten different examples of molecular scaffolds that inhibit bacterial proliferation through targeting FtsZ and that represent promising antibacterial leads are described below.

### 2.2. FtsZ Inhibitors

#### 2.2.1. Benzamides

**Figure 3:**
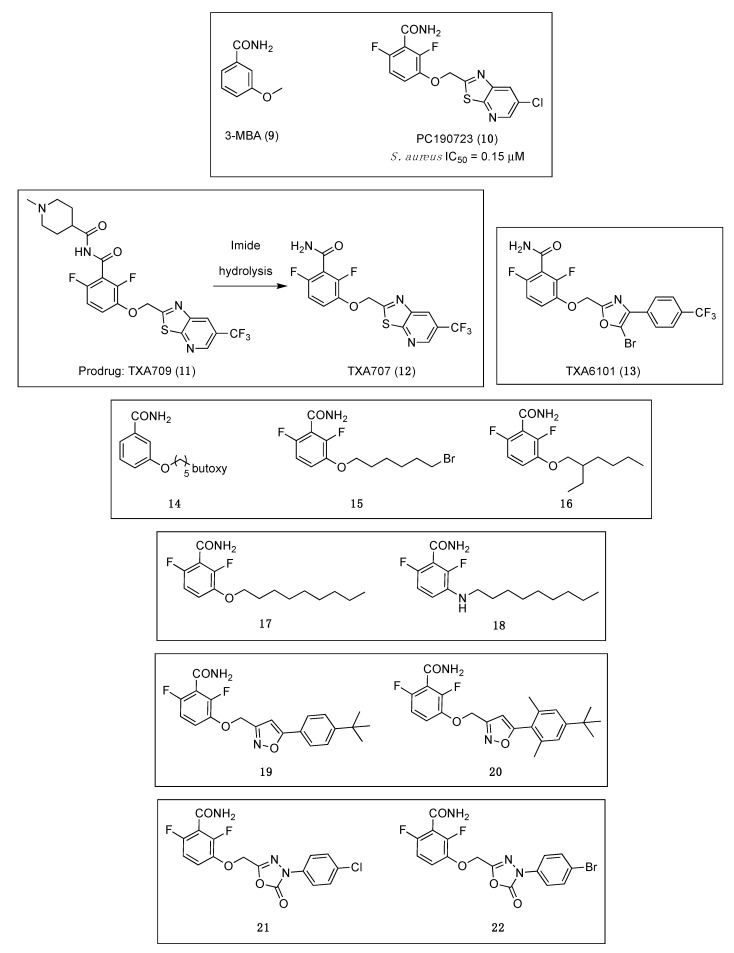
Chemical structures of the benzamide and structurally related derivatives.

A fragment-based drug discovery program to improve the ligand 3-methoxybenzamide (3-MBA, (**9**), Figure 3) [36], resulted in the FtsZ inhibitor PC190723 (Figure 3 (**10**)), a compound that combined a substituted benzamide with a thiazolopyridine moiety by means of an ether linker. PC190723 is a benzamide that has potent antibacterial activity against *B. subtilis* and several strains of *S. aureus*, which opened the avenue towards the discovery of a new class of small molecules that target FtsZ and inhibit cell division [37]. Even though it was inactive against a number of Gram-negative strains, this benzamide derivative was able to inhibit the growth of *B. subtilis* and several species of *Staphylococci*, including MRSA and multidrug-resistant *S. aureus* (MDRSA), with minimum inhibitory concentration (MIC) values ranging from 1.41 to 2.81 µM (Table 1) [38]. The mode of action of PC190723 was confirmed by means of an in vitro assay in which it was found that it is able to inhibit the GTPase activity of *S. aureus* FtsZ (*Sa*FtsZ) in a concentration-dependent manner (IC_50_ = 0.15 µM). One of the distinctive characteristics of FtsZ-interacting compounds is the induction of elongation or enlargement of treated bacterial cells. In line with this, the studies of the morphology of the cells after treatment with PC190723 revealed that the benzamide derivative causes the elongation of rod-shaped *B. subtilis* and the enlargement of spherical *S. aureus*.

**Figure 4:**
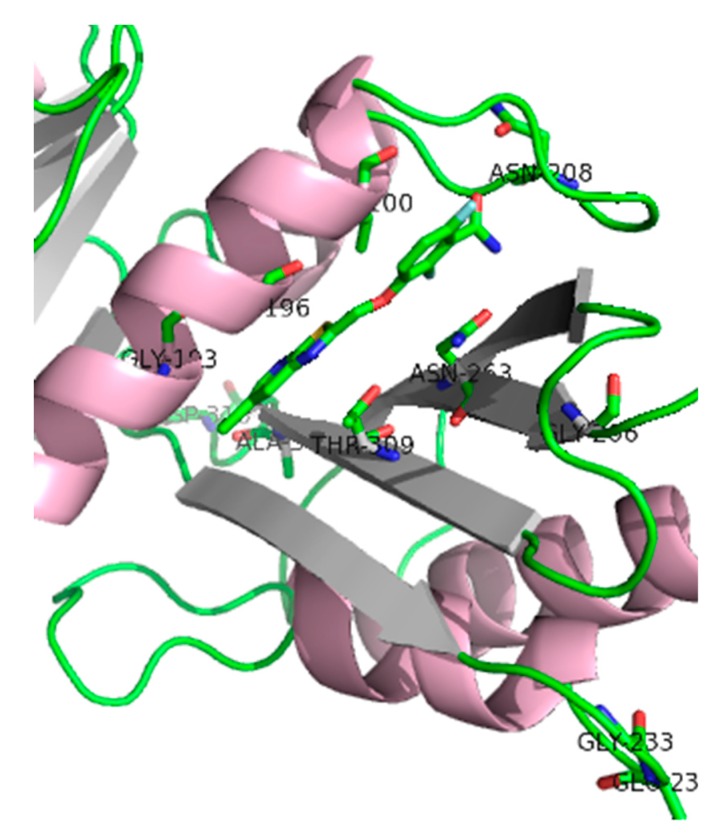
*Sa*FtsZ in complex with benzamide PC190723 (**10**) (PDB entry 4DXD). Residues for which amino acid substitution resistance mutations have been identified are highlighted. It is worth noting that Gly^193^ and Gly^196^ residues account for about 90% of the resistant isolates [33].

Crystallography studies (2.01 Å) of *S. aureus* FtsZ-GDP in complex with PC190723 revealed that the ligand binds to a narrow and hydrophobic pocket within the deep cleft formed by the C-terminal half of the H7 helix, the T7 loop, and the C-terminal four-stranded β sheet [27,33]. The difluorobenzamide and the thiazolopyridine moieties are located in the same plane, and the benzamide interacts directly with the T7 loop (Figure 4).

PC190723 drug resistance mapping analysis of PC190723-resistant isolates yielded resistant clones at a frequency of resistance (FOR) of 3 × 10^−8^, which is borderline for its development as a single-agent antibacterial. The genetic analysis revealed that the clones contain an amino acid change at one of the following residues F40L, E90K, Q94L, N170K, G193D, G196A, G196C, G196S, G196V, L200F, L200I, N208D, G233V, E234K, N263I, N263K, N263Y, G266D, G266V, T309I, A312E, D316E, and T329A [33]. Such genetic studies, in which mutations in the target lead to resistance, are key for validating a specific bacterial target and confirm on-targets effects. Additionally, these data are important to anticipate and minimize any potential resistance issues [39].

One of the most widely used strategies to enhance antibiotic efficacy and fight antibiotic resistance consists of the combination of antibiotics with a second agent. This synergistic approach includes very well-known approved examples such as the association of an antibiotic (i.e., amoxicillin, ampicillin, piperacillin) with a β-lactamase inhibitor (i.e., clavulanic acid, sulbactam, tazobactam). PC190723 resistance issues led Tan and co-workers to investigate its combination with a β-lactam antibiotic. The researchers found excellent synergistic effects when PC190723 was used in combination with imipenem (at 13.36 μM) against fifty-three MRSA strains, with submicromolar MIC values of ≤0.70 μM [33]. Remarkably, this association also led to a substantial reduction of the FOR from 3.0 × 10^−8^ to 1.6 × 10^−9^.

In spite of all these advancements, the suboptimal pharmaceutical and pharmacokinetic properties of PC190723 impeded its clinical development. Recently, the replacement of the chlorine atom on the pyridyl ring of PC190723 by a metabolically more stable CF_3_ group led to the identification of benzamide TXA707 (Figure 3 (**12**)) as a promising FtsZ-targeting agent with not only improved metabolic stability and pharmacokinetic properties, but also superior in vivo anti-staphylococcal activity than PC190723 (**10**) [40]. The antibacterial activity of TXA707 (**12**) was evaluated against a panel of eighty-four clinical *S. aureus* isolates, including methicillin-resistant, vancomycin-intermediate, vancomycin-resistant, daptomycin-non susceptible, linezolid-non susceptible, and methicillin-susceptible *S. aureus* strains (modal MIC value = 2.57 μM). In order to establish that TXA707’s antibacterial activity occurs through inhibition of FtsZ, the authors studied (i) its impact on FtsZ polymerization; (ii) its effect on FtsZ Z-ring formation; (iii) its impact on septum formation and cell division; and (iv) the resistance mutations. Similar to PC190723, TXA707 stimulates FtsZ polymerization in a concentration-dependent manner and provokes an enlargement of *S. aureus* cells (approximately 3-fold, compared to control cells). Resistance mutation analysis of benzamide TXA707 was also studied. A large-inoculum strategy yielded resistant clones at a FOR of 3 × 10^−8^ in MRSA ATCC 43300 and 1 × 10^−8^ in an MRSA clinical isolate. Sequencing and analysis of the clones determined that the most prominent mutation is the G196S mutation (55%), followed by a G193D mutation (15%), an N263K mutation (15%), a G196C mutation (10%) and a G196A mutation (5%). Furthermore, Kaul et al. demonstrated that TXA707 prodrug (TXA709 (**11**)**,** Figure 3) is also both orally and intravenously efficacious in vivo in mouse models of methicillin-sensitive *S. aureus* (MSSA) and MRSA infection, and that it exhibits minimal toxicity to mammalian cells (i.e., HeLa and MDCK cells), with IC_50_ values >233.25 μM.

TXA707 (**12**) and its prodrug TXA709 (**11**), which are currently being developed by TAXIS Pharmaceuticals, display a superior potency and pharmacokinetic profile that provide evidence of the potential of the benzamide scaffold for its development as an anti-staphylococcal clinical candidate.

**Figure 5:**
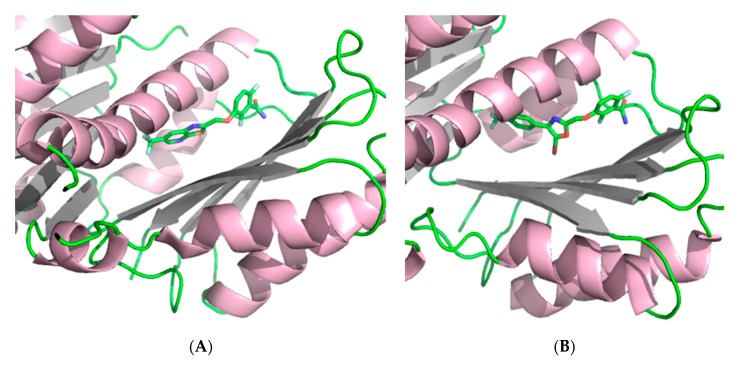
(**A**) Crystal structure of *Sa*FtsZ at 1.3 Å in complex with TXA707 (**12**) (PDB entry 5XDT) [41]. (**B**) Crystal structure of *Sa*FtsZ at 2 Å in complex with TXA6101 (**13**) (PDB entry 5XDU) [41].

The observation of mutations in *Sa*FtsZ that confer resistance to TXA707 incited the development of the more structurally flexible benzamide TXA6101 (Figure 3 (**13**)), which retained antibacterial activity against MRSA isolates that express either of the two most prevalent mutations (i.e., G193D or G196S) [41]. The difluorobenzamide moiety is common in TXA6101 and TXA707, but they differ in the rotational flexibility of the rings at the opposite end. The oxazole and the phenyl ring in TXA6101 are linked by a single bond enabling the free rotation of both rings. The antibacterial activity of TXA6101 against an MRSA clinical isolate is almost 10-fold better than that of TXA707 (MIC = 0.26 and 2.57 μM, respectively). TXA6101 also exhibited a remarkable antibacterial activity of 2.09 μM against MRSA MPW020 expressing either G193D and G196S mutant FtsZ and the FOR value against the aforesaid isolate was determined to be 3.6 × 10^−9^, 10-fold better than that for TXA707 (i.e., 4.3 × 10^−8^). Crystallography studies of TXA6101 (**13**) in complex with wild-type *S. aureus* revealed that it binds to a similar pocket to TXA707 (**12**) and PC190723 (**10**), but it adopts a bent conformation similar to TXA707. The binding of TXA707 and TXA6101 induces the conformational rearrangement of I197, M226, and I311 that leads to the formation of an inner hydrophobic pocket, with M226 acting as a gate that opens the pocket (Figure 5).

In 2017, inspired by the promising results displayed by 3-MBA and PC190723, Bi et al. generated a series of 3-*O*-arylalkyl-2,6-difluorobenzamide derivatives [42]. Using benzamide **14** (Figure 3) as a starting point for the discovery of new FtsZ inhibitors, fluorine atoms were introduced at positions 2 and 6 of the benzene ring, and the 3-*O*-arylalkyl side chain diversified with varied groups such as alkyl halides, branched alkyl groups, esters, and heterocycles. Among the compounds tested, compounds **15** and **16** (Figure 3), bearing a 3-bromoalkoxy and a 3-alkyloxy side chain, respectively, displayed the best antibacterial activity against the tested Gram-positive species such as *B. subtilis* and different species of *Staphylococci* (including MRSA ATCC 2923) with MICs ranging from 0.88 to 28.04 μM (Table 1). Regrettably, all the derivatives displayed no activity against Gram-negative strains (i.e., *E. coli* ATCC 25922 and *P. aeruginosa* ATCC 27853), most probably due to their inability to penetrate the bacterial cell. The cell division inhibitory assay followed the same trend as that observed in the antibacterial tests: benzamides **15** and **16** showed potent cell division inhibitory activities against *B. subtilis* ATCC 9372 and *S. aureus* ATCC 25923. The results were particularly outstanding on rod-shaped *B. subtilis* for which filamentation was observed at concentrations as low as 1.49 μM and 0.44 μM after exposure to **15** and **16**, respectively. It is worth highlighting that, although enlarged morphology is one of the hallmarks of FtsZ-targeting compounds, phenotypic studies combined with antibacterial activity alone do not necessarily validate on-target effects.

Recently, Lui et al. reported an elegant study in which a focused-compound library of forty-seven 3-aminobenzamides and structurally related derivatives was generated using benzamides **17** [43] and PC190723 (**10**) [44] as templates. Their investigation successfully led to the identification of the *n*-nonylamino benzamide **18** (Figure 3), which was able to inhibit the growth of *S. aureus* with a MIC value of 3.35 µM (Table 1) [45]. Afterward, the authors evaluated the synergistic effects of **18** in combination with diverse β-lactam antibiotics in clinical use, including the penicillin-type antibiotics methicillin (ME), cloxacillin (CL), and amoxicillin (AM), the cephalosporin-type antibiotic cefuroxime (CX), and the carbapenem-type antibiotic meropenem (MR), against a panel of twenty-eight clinically relevant strains of MRSA. As shown in Table 2, the combination of **18** with the different β-lactam antibiotics led to an enhancement of its antibacterial potency against three of the MRSA isolates, synergistic effects that reflect its potential as an adjuvant in antibiotic combination therapy. Computational studies revealed that, like benzamide PC190723, **18** also binds directly into the cleft between H7 and the C-terminal domain of *S. aureus* FtsZ. Afterward, Lui et al. used in vitro light scattering assays to investigate the effect on the FtsZ polymerization upon treatment with **18**. Interestingly, the authors found that **18**, unlike PC190723 (**10**), potently inhibits FtsZ polymerization in a concentration-dependent manner. Transmission electron microscopy (TEM) imaging revealed that, upon treatment with **18**, the heavily dense network of *S. aureus* FtsZ filaments becomes noticeably short and thin. A result that corroborates the light scattering assay results and that suggests that benzamide **18** is able to inhibit FtsZ polymerization.

Bi et al. utilized a combination of docking studies and structure-based optimization strategies to design and synthesize two series of benzamide derivatives containing an isoxazol scaffold in their structure [46]. Antibacterial evaluation of the compounds against a panel of Gram-positive and Gram-negative pathogens led to the identification of the isoxazol-5-yl-3-benzamide derivatives **19** and **20** (Figure 3), which displayed excellent antibacterial and FtsZ inhibition activities. In particular, **19** showed remarkable MIC values of 0.08 and 0.04 μM against *B. pumilus* and *B. subtilis*, respectively (Table 1). Notably, both of the derivatives were also able to inhibit the growth of one susceptible and two resistant *S. aureus* strains with MIC values ranging from 2.41 to 10.35 μM, concentrations that are comparable to the reference antibiotic linezolid on susceptible *S. aureus* (MIC = 6 μM). Like in other studies, none of the derivatives were able to inhibit the growth of the two Gram-negative microorganisms tested (i.e., *E. coli* ATCC 25922 and *P. aeruginosa* ATCC 27853). Afterward, the authors took benzamide **19** further and studied its impact on the bacterial cell morphology of *B. pumilus* by means of microscopic observations. Compound **19** was able to increase the cell length of this microorganism at 0.04 µM, a result that is consistent with other benzamides previously reported [36,42], and which, therefore, suggests that **19** interferes with the FtsZ function, leading to abnormal cell division and eventually cell death. The in vitro evaluation of the impact of benzamide **19** on *B. subtilis* FtsZ revealed that it stimulates *B. subtilis* FtsZ (*Bs*FtsZ) in a concentration-dependent manner. Visualization of *Bs*FtsZ polymers by means of TEM showed that the presence of this benzamide at 25.88 µM increases the size and the thickness of the FtsZ polymers and the bundling of the FtsZ protofilaments. Computational studies were carried out to predict the binding mode of **19**, which revealed that the nitrogen atom of the isoxazol forms a unique ion-dipole interaction with the carboxyl group of an aspartate residue (D199) that justifies the superior antibacterial activity of this derivative. These results, together with its minimal toxicity on HeLa cells (IC_50_ > 331 μM), emphasize the potential of this benzamide derivative as a lead compound and pave the way towards the discovery of new benzamide FtsZ inhibitors as antibacterial agents.

Recently, in a continuation of their work on FtsZ inhibitors, Bi et al. reported the design, the synthesis, and the antibacterial activity of six series of benzamide derivatives bearing a 1,3,4-ozadiazol-2-one moiety in their structure [47]. Among them, the authors identified benzamides **21** and **22** (Figure 3), which possess strong antibacterial activity against several Gram-positive bacteria with MIC values in the range of 0.29–5.24 μM (Table 1). Notably, both of the derivatives are able to inhibit the growth of drug-resistant *S. aureus* isolates with minimum inhibitory concentrations ranging from 2.35–5.24 μM. Microscopic observations determined that compound **22** is able to significantly increase the cell length of *B. pumilus*, a result that is consistent with other previously studied benzamide derivatives. *Bs*FtsZ dynamic studies showed that benzamide **22** stimulates the protein polymerization in a concentration-dependent fashion. Additionally, **22** was also found to be minimally toxic against HeLa cells with an IC_50_ > 150.17 μM, which is significantly higher than the antibacterial MIC values. This derivative was also chosen for docking analysis with *Sa*FtsZ, a study that indicated that **19**, like benzamide PC190723 (**10**), binds into the pocket formed by the T7 loop, the H7 helix, and the C-terminal sheet. 

All of the abovementioned results provide an encouraging opportunity to guide research around the benzamide moiety as a promising scaffold for the development of new antibacterials that target FtsZ.

#### 2.2.2. Quinolinium and Structurally Related Compounds

**Figure 6:**
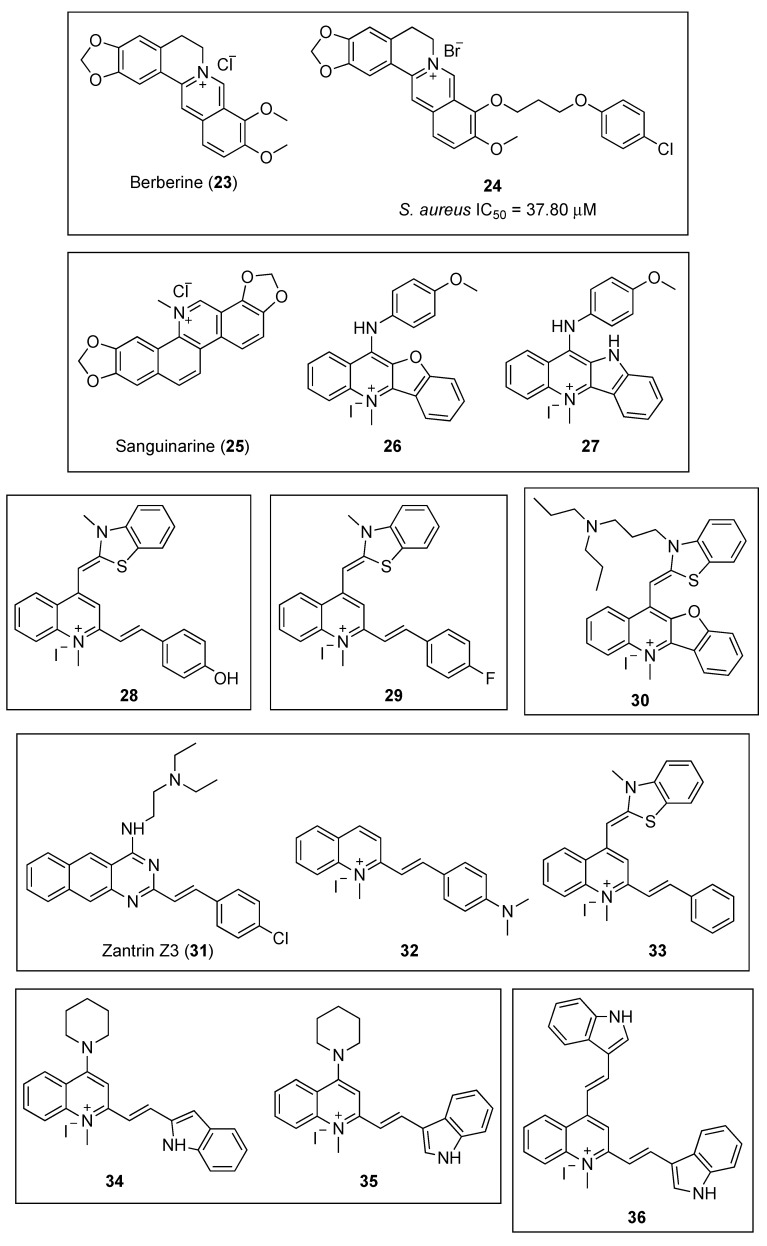
Chemical structures of the quinolinium and structurally related compounds.

The plant alkaloid berberine (Figure 6, (**23**)) has been the object of numerous studies aimed to determine that it exhibits antibacterial properties by targeting the cell division protein FtsZ; however, conflicting results have been reported. Biochemical and genetic studies by Domadia and Boberek argued for FtsZ inhibition as the primary target for berberine in bacteria [48,49], while more recent fluorescence anisotropy studies have not supported this hypothesis and concluded that the observed fluorescence was due to the intrinsic auto-fluorescence of the alkaloid [32].

By means of in silico structure-based design, together with antibacterial evaluation, Sun and co-workers identified the 9-phenoxyalkyl berberine derivative **24** (Figure 6) as a potent inhibitor of the growth of Gram-positive microorganisms (Table 3), with MIC values between 3.50 and 7.01 µM, including methicillin-resistant *S. aureus* and vancomycin-resistant *E. faecium* [50]. Afterward, the evaluation of the effect of this derivative on the GTPase activity of *Sa*FtsZ showed that the berberine analogue **24** is able to potently inhibit its activity (IC_50_ = 37.80 μM), a result that correlates well with the antibacterial tests. Light scattering studies and transmission electron microscopy revealed that **24** inhibits *Sa*FtsZ polymerization in a dose-dependent manner. Finally, microscopy observations of *B. subtilis* cell morphology concluded that compound **24** is able to elongate the microorganism cell length compared with untreated cells. It is worth highlighting that similar results were obtained with FtsZ inhibitors of a different chemotype, such as the benzamide PC190723 (Figure 3, (**10**)) [37].

Sun et al., taking natural products berberine (**23**) and sanguinarine (**25**) (Figure 6) as a lead, undertook the design and synthesis of a novel library of N-methylbenzofuro [3,2-*b*] quinoline and N-methylbenzoindolo [3,2-*b*] quinoline derivatives, which contained a quaternary pyridinium center [51]. Pleasingly, the benzofuroquinolinium **26** and the benzoindoloquinolinium **27** (Figure 6) exhibited significant activity against several Gram-positive and Gram-negative pathogens, which included *B. subtilis*, *S. aureus*, *E. faecium*, *E. coli*, *P. aeruginosa,* and *K. pneumoniae* (Table 3). Remarkably, compound **27** displayed a MIC value against vancomycin-resistant *E. faecium* superior to that of the reference antibiotics methicillin and vancomycin (MIC values of 16 and 44 μM, respectively). In addition to the antibacterial screening, the authors also investigated the effects of both of these compounds on the polymerization of purified *Sa*FtsZ. Light scattering assays yielded that both **26** and **27** produced an evident inhibition of the *Sa*FtsZ at 33.24 µM. The study of the effects **27** on the GTPase activity of *Sa*FtsZ revealed that this compound is able to inhibit the activity in a dose-dependent manner. Furthermore, microscopic observations of *B. subtilis* cell morphology showed that **27** increases the cell length of this microorganism, leading to an abnormal cell division and, ultimately, cell death. These results corroborate that chemotypes that interfere with the GTPase activity and FtsZ polymerization are promising starting points for the development of novel antibacterial agents.

In 2017, the same authors identified, by cell-based screening, a potent cell division inhibitor, the thiazole orange derivative **28** (Figure 6) [52]. Sun et al. screened compound **28** against a panel of twenty-five clinically relevant bacterial strains, and the results revealed that this quinolinium derivative is able to inhibit the growth of nearly all the strains tested, with MIC values ranging from 1.36 to 5.45 µM for the Gram-positive bacteria, and from 5.45 to 87.20 µM for the Gram-negative microorganisms (Table 3). It is worth highlighting the excellent antibacterial profile of **28** on species of *Staphylococci* spp. (MIC = 1.36–5.45 μM, including MRSA species), on *E. coli* and its drug-resistant strain (MIC = 5.45 μM), and on other Gram-negative species such as *P. aeruginosa* with a moderate minimum inhibitory concentration of 10.90 µM. Moreover, light scattering results reflected that **28** is able to stimulate *Sa*FtsZ and *E. coli* FtsZ (*Ec*FtsZ) in a concentration-dependent manner. Furthermore, the study of its inhibitory effect on the GTPase activity of both of the FtsZ proteins showed that **28** decreases the rate of GTP hydrolysis also in a concentration-dependent fashion (58% inhibition at 14.53 µM). All of the above, together with its lack of toxicity against mammalian cells L929 and HK-2 with IC_50_ values of 96.50 and 98.15 μM, respectively, make it an attractive hit for the development of other antibacterial FtsZ-targeting agents based on the quinoline scaffold.

In a continuation of their work on the identification of novel anti-FtsZ agents, Sun and co-workers reported the design, synthesis, and antibacterial activity of a new series of 3-methylbenzo [*d*] thiazol-methylquinolinium derivatives [53]. Among the sixteen compounds, the quinolinium **29** (Figure 6) displayed the best antibacterial profile against drug-sensitive and drug-resistant Gram-negative strains with MIC values lower than 5.43 and 10.86 µM, respectively (Table 3), efficacies on drug-resistant strains that are comparable to those of the clinical antibiotic methicillin (MIC values >16 μM). In addition, it is worth emphasizing the potent activity it exhibited against MRSA, with a MIC value as low as 2.72 μM. Afterward, the study of its mode of action revealed that quinolinium **29** inhibits GTP activity and stimulates polymerization of *Sa*FtsZ in a concentration-dependent fashion. Morphology studies showed that treatment with compound **29** caused a 2- to 5-fold enlargement of *B. subtilis* cells, whose cell lengths increased from 2–10 μm to longer than 20 μm. Additionally, this derivative was found to be minimally toxic against mammalian cell lines HK-2 and L929, with IC_50_ values of 78.25 and 82.74 µM, respectively, which are significantly higher than the reported MIC values. Molecular modeling was used to identify the binding site of **29** in FtsZ. A 2.01 Å crystal structure of *Sa*FtsZ showed that the ligand binds near the T7-loop and the H-7 helix, and that the cationic quinolinium moiety is predicted to interact with the negatively charged residue D199.

In 2018, Zheng et al. reported the repurposing of a small collection of G-quadruplex fluorescent probes as inhibitors of the polymerization of FtsZ [54]. All the benzofuroquinolinium derivatives possessed broad and potent antibacterial activity against the tested strains, including drug-resistant bacteria. Among them, benzofuroquinolinium **30** (Figure 6) exhibited the best values with MIC values in the submicromolar range for Gram-positive species such as *B. subtilis* 168, *S. aureus* ATCC 29213 and MRSA ATCC 33592 (Table 3). This compound also showed very promising antibacterial results against Gram-negative strains such as *E. coli* ATCC 25922, *A. baumannii* ATCC 19606, *P. aeruginosa* ATCC BAA-2108, and *K. pneumoniae* ATCC BAA-2470 with MIC values of 1.54, 12.31, 6.16, and 12.31 μM, respectively. The study of the effect of **30** on the morphology of *B. subtilis* showed an elongation of the cell lengths from 5–10 to 20 μm. Likewise, the treatment of *E. coli* with **30** produced a 3- to 6-fold increase of the cell lengths (i.e., from 2–4 µm of normal cells to longer than 12 μm after treatment with **30**). In line with previous reports on FtsZ inhibitors, **30** was able to inhibit the GTPase activity and the polymerization of *Ec*FtsZ in a concentration-dependent manner. Computational studies predicted that **30** binds to the *Sa*FtsZ GTP binding pocket where its dipropyl moiety is involved in additional and crucial hydrophobic interactions with several residues that lie within this pocket. Furthermore, **30** displays minimal toxicity on mammalian cells (i.e., HK2, L929, and HepG2), and therefore, constitutes a promising new scaffold for the development of novel antibacterial agents targeting FtsZ.

Inspired by the finding that Zantrin Z3 (**31**) [55] and quinolinium **32** (Figure 6) inhibit the proliferation of Gram-positive microorganisms, Fang et al. screened the 1-methylquinolinium derivative **33** (Figure 6) against a panel of clinically relevant strains and studied its synergistic effects in combination with β-lactam antibiotics [56]. The antibacterial evaluation results showed that **33** is able to inhibit the growth of not only Gram-positive drug-sensitive strains with MIC values ranging from 1.40 to 3.74 μM, but it also displayed a strong inhibitory effect against the tested MRSA with MIC values as low as 2.81 μM (Table 3). In the case of Gram-negative microorganisms, **33** showed a noteworthy antibacterial profile against *E. coli* and *P. aeruginosa* (MIC values of 5.61 and 11.23 µM, respectively). In order to carry out the study of the synergistic effects, the authors chose different β-lactam antibiotics such as ampicillin, methicillin, oxacillin, imipenem, and ceftazidime (Table 4). Remarkably, compound **33** improved the antibacterial activity of all the β-lactam antibacterials tested. It is worth highlighting the 4-fold enhancement in the antibacterial activity of both ampicillin against an ampicillin-resistant *S. aureus* strain and of imipenem against the MRSA strain ATCC BAA-41.

The study of the effects of **33** in the morphology of *B. subtilis* revealed that the cells were significantly elongated as its lengths were found to be longer than 20 µm. A phenomenon that could also be observed in previous studies with other quinoline-based FtsZ inhibitors. Finally, by means of biochemical studies, the authors determined that quinolinium **33** is able to inhibit the GTPase activity and enhance the polymerization of *Sa*FtsZ in a dose-dependent manner.

Recently, in 2019, Cai et al. explored the introduction of an aromatic heterocyclic moiety into the quinolinium scaffold and developed a series of indolyl quinolinium derivatives whose antibacterial activity and mode of action were evaluated [57]. Among the fifteen indolyl quinolinium derivatives, compounds **34** and **35** (Figure 6), bearing a piperidine moiety at position 4 of the quinolinium core, exhibited the best antibacterial profile with MIC values against Gram-positive strains ranging from 2.02–8.07 µM (Table 3). These compounds also showed moderate antibacterial activity against microorganisms with MIC values against *E. coli* ATCC 25922 as low as 32.30 and 16.15 µM, respectively. The authors also investigated the effect of the compounds on *B. subtilis* cell morphology. Remarkably, indolyl quinolinium **34** was able to increase the cell length from the range of 5–13 µm to 70–130 µm. The interferential effects of **35** on the GTPase activity and the polymerization of FtsZ revealed that it is able to disrupt both of the processes in a concentration-dependent manner. In agreement with previous studies, the molecular modeling analysis predicted that derivative **35** binds to a hydrophobic and narrow cleft formed by helix H7, loop T7, and a four-stranded β-sheet. Similar to quinolinium **29**, the cationic quinolinium moiety is predicted to interact with a negatively charged aspartate residue (D199).

Sun et al. evaluated the antibacterial activity of the 1-methylquinolinium derivative BIMQ (**36**, Figure 6) [58]. Notably, it displayed an excellent antibacterial profile against the tested Gram-positive microorganisms with MIC values in the range of 1.81–3.61 µM (Table 3). In addition, this quinolinium derivative exhibited a strong antibacterial activity against several strains of *S. aureus* (which included an MRSA isolate) and *E. faecium* ATCC 49624 with minimum inhibitory concentrations as low as 1.81 µM. Nonetheless, **36** showed a more moderate potency against Gram-negative microorganisms (MIC = 14.45–115.64 µM). The study of the morphology of *B. subtilis* cells revealed that the typical short rod morphology of the bacillus, with cell lengths of 8–12 µm, was significantly elongated after treatment with BIMQ. The researchers also determined that quinolinium **36** is able to increase the GTPase activity and enhance the polymerization of FtsZ in a concentration-dependent manner. Furthermore, the molecular docking study predicted that BIMQ binds into the cleft situated in between the C-terminal domain, the H-7 helix, and the T-7 loop, a result that is in agreement with that of other FtsZ inhibitors such as the benzamide PC190723 (Figure 3, (**10**)).

#### 2.2.3. Dihydroquinolines

**Figure 7:**
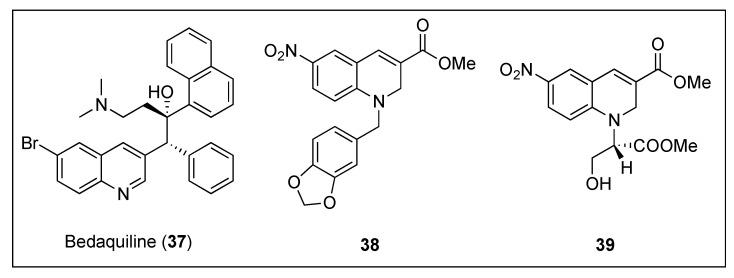
Chemical structures of bedaquiline (**37**) and 1,2-dihydroquinolines **38** and **39**.

The development of novel therapeutic agents against *Mycobacterium tuberculosis* that interfere with its cytokinesis process has also caught the attention of research groups around the world. Taking anti-tubercular drug bedaquiline (Figure 7, (**37**)) as a lead, Duggirala and co-workers reported the synthesis and biological evaluation against mycobacteria of a collection of 1,2-dihydroquinolines [59]. The most promising compounds, i.e., **38** and **39** (Figure 7), were able to inhibit the growth of *Mycobacterium smegmatis* with MICs ranging from 2.58–22.30 µM (Table 5). Afterward, **39** was evaluated on its ability to alter the morphology of *M. smegmatis* and *B. subtilis* cells. In line with previous studies, in the presence of dihydroquinoline **39,** the length of the mycobacterium increased approximately by 4-fold (from 6.40 to 22.96 µm), whilst treatment of the bacillus with **39** led to a 6-fold elongation of its cell length. Light scattering studies confirmed that these compounds also inhibit the polymerization of *Mtb*FtsZ with IC_50_ values as low as 33.40 µM for dihydroquinoline **39**. Additionally, they were found to inhibit its GTPase activity in a dose dependent manner and within similar IC_50_ values (Table 5). Having observed that several antibiotics, such as rifampicin or quinolones, are able to induce oxidative stress in bacteria, in addition to their target-specific effects, the authors also quantified the levels of ROS (reactive oxygen species) generated under stress conditions by means of a fluorescence spectrometer. Intracellular ROS studies of *M. smegmatis* cells after treatment with **39** revealed a DCF-induced fluorescence, indicating that dihydroquinoline **39** is able to induce ROS in this species. Since ROS can activate the SOS response in bacteria, which results in cell elongation, in order to exclude the possibility that this may be the cause of the morphology change and to verify in vivo binding to FtsZ, several assays including Z-ring localization and X-ray crystallography of the dihydroquinoline in complex with the protein, would need to be carried out.

#### 2.2.4. Phenanthridium Derivatives

**Figure 8:**
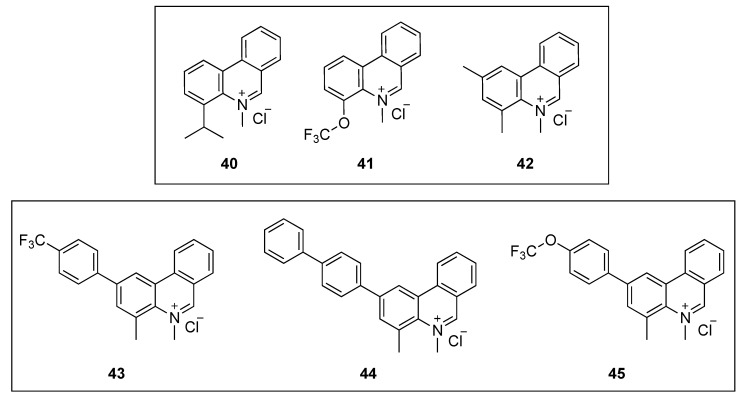
Chemical structures of the phenanthridium derivatives.

Liu and co-workers designed, synthesized, and evaluated the antibacterial activity of a collection of 5-methylphenanthridium derivatives [60]. Among them, compounds **40**, **41**, and **42** (Figure 8) exhibited promising antibacterial activities against *B. subtilis* ATCC 9372 and *S. pyogenes* PS (MIC = 12.75–15.52 µM). Remarkably, phenanthridium **42** also displayed strong activity against a penicillin-resistant strain of *S. aureus* (Table 6). Unfortunately, none of the derivatives were able to inhibit the growth of the two Gram-negative bacteria (i.e., *E. coli* ATCC 25922 and *P. aeruginosa* ATCC 27853). Afterward, phase-contrast light microscopy was used to carry out the cell morphology studies. All the compounds were able to cause filamentation of *B. subtilis* or ballooning of *S. aureus* at their respective MIC values. The effect of the phenanthridium derivatives on the polymerization of mammalian tubulin was also evaluated in vitro. None of the compounds enhanced the polymerization even at concentrations higher than that of the positive control paclitaxel. It is worth noting that, although it is important to assess off-target effects on FtsZ homolog tubulin, evaluating these together with morphologycal studies alone, could, unfortunately, result in a false-positive FtsZ inhibitor.

In a continuation of their work on the development of FtsZ inhibitors based on the phenanthridium scaffold, Liu et al. identified three phenanthridium derivatives (**43**, **44,** and **45**, Figure 8) that displayed an excellent antibacterial profile, potent cell division activity, and noticeable inhibition of *Bs*FtsZ polymerization [61]. Promisingly, the three compounds exhibited outstanding antibacterial activities against the Gram-positive strains tested (Table 6). Remarkably, compound **43** displayed MIC values as low as 0.15 µM against *B. subtilis* ATCC 9372, *S. pumilus* ATCC 63202, and *S. aureus* ATCC 25923, which were at least 40-fold superior to that of sanguinarine and reference drugs such as ciprofloxacin and oxacillin (MIC values = 6–72 µM). The cell division inhibitory activity using a light microscope revealed that 2-phenylphenanthridium **43** increases the length of *B. subtilis* and enlarges *S. aureus*, compared to untreated cells. Although the cell morphology studies were investigated using **43** as a representative compound of this collection of phenanthridium derivatives, the authors selected compound **44** to assess the effect of the series on the dynamic polymerization of *Bs*FtsZ by means of a light scattering assay. Phenanthridium **44** was able to inhibit the protein polymerization at 12.50 µM, and, in line with previous reports, in a dose-dependent manner. Finally, molecular modeling studies of **42** on *Bs*FtsZ showed that this phenanthridium derivative binds to the narrow cleft delimited by the H-7 helix, the T-7 loop, and the C-terminal subdomain. A binding mode that is similar to that of the benzoindoloquinolinium **27** (Figure 6) [51].

#### 2.2.5. Quinuclidines

**Figure 9:**
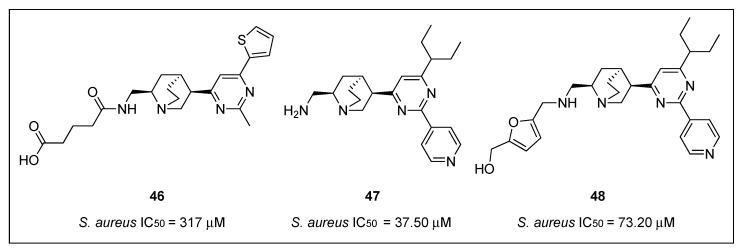
Chemical structures of the quinuclidine derivatives.

Structure-base virtual screening of a library of approximately 20,000 compounds from Analyticon Discovery against the crystal structure of *Methanococcus jannaschiii* FtsZ led to the identification of the pyrimidine-substituted quinuclidine **46** (Figure 9) as a new class of FtsZ inhibitors with moderate antibacterial activity [62]. Afterward, an additional cycle of virtual screening of a library of nearly 700 pyrimidine-quinuclidine scaffolds from the same source conducted to quinuclidines **47** and **48** (Figure 9), which were able to inhibit in vitro the proliferation of not only Gram-positive *S. aureus*, but also Gram-negative *E. coli* (Table 7), with MIC values ranging from 24.60 to 75.70 µM. Additionally, the authors determined that quinuclidine **47** is able to potently inhibit the FtsZ assembly without affecting the activity of mammalian tubulin, an encouraging finding for the development of novel quinuclidine-based FtsZ inhibitors as antibacterial agents.

In a continuation of their work on the investigation of the underlying mechanisms of antibacterial activity of these novel FtsZ inhibitors, the research group studied the synergistic effects of quinuclidine **48** in combination with β-lactam antibiotics against antibiotic-resistant strains of *S. aureus* [63]. Firstly, compound **48** was tested against a panel of clinically relevant bacterial species, including antibiotic-susceptible strains (e.g., *B. subtilis* 168, *E. faecium* ATCC 49624, and *E. faecalis* ATCC 29212) and antibiotic-resistant strains (e.g., ampicillin-resistant *S. aureus* ATCC 29247, methicillin-resistant *S. aureus* ATCC BAA-41, multidrug-resistant *S. aureus* ATCC BAA-44, and vancomycin-resistant *E. faecium* ATCC 700221). Quinuclidine **48** was able to inhibit the growth of all the microorganisms tested (Table 7), an outcome that indicates its favorable antibacterial profile against both antibiotic-susceptible and antibiotic-resistant strains. Afterward, the group studied the combination of **48** with other β -lactam antibiotics, such as ampicillin (AP), oxacillin (OX), methicillin (ME), imipenem (IM), cefoxitin (CF), and ceftazidime (CZ) (Table 8). These assays revealed that quinuclidine **48** is able to cause a four- and eight-fold improvement of the antibacterial activity of imipenem and ceftazidime, respectively (MIC values decreased from 53.45 to 13.36 µM and from 58.55 to 7.32 µM). Results that are in good agreement with the work previously reported by Tan et al., in which they determined the synergistic effects of benzamide PC190723 (**10**) with β-lactam antibiotics against MRSA strains [33]. Light scattering studies revealed that **48** slowed down the in vitro assembly and the bundling of FtsZ monomers in a dose-dependent manner. Quinuclidine **48** was also able to disrupt the formation of the Z-ring in *E. coli*. After treatment with **48**, the percentage of cells having an integral Z-ring dramatically diminished from 93% (at 0 µM) to 24% (at 50 µM). The results of this study have certainly paved the way for the development of other quinuclidine derivatives as a promising new class of FtsZ-targeting compounds with antibacterial activity.

#### 2.2.6. Pyrimidines

**Figure 10:**
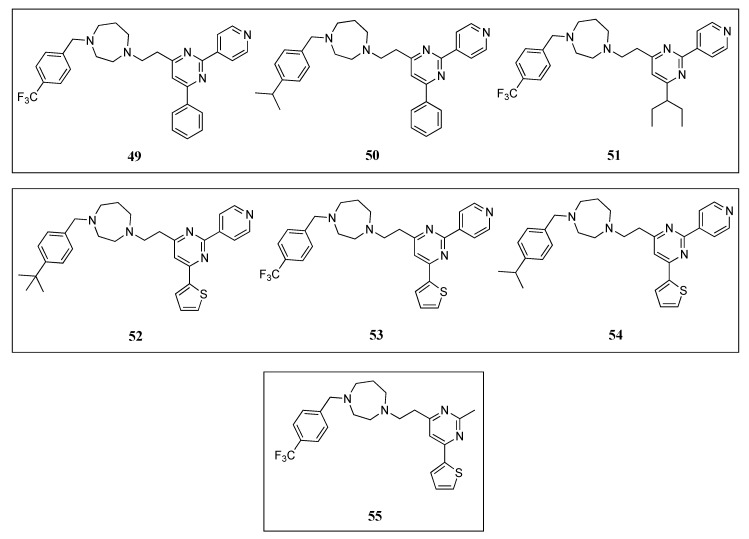
Chemical structures of the pyrimidine derivatives.

Molecular docking studies of quinuclidines **47** and **48** (Figure 9) and GTP molecules using *Sa*FtsZ revealed that the 2,4,6-trisubstituted pyrimidine moiety is crucial for binding [62]. Encouraged by this finding, Chan and co-workers envisaged the design of a molecule that retained the pyrimidine moiety, but in which the chiral quinuclidine was replaced by a less structurally complex and more synthetically accessible amine scaffold, a strategy that successfully led to the identification of a new class of FtsZ inhibitors based on the 2,4,6-trisubstituted pyrimidine scaffold [64]. Among the ninety-nine compounds of this new library, pyrimidines **49**, **50**, and **51** (Figure 10) displayed the best antimicrobial activities against the Gram-positive *S. aureus* ATCC 29213 and the Gram-negative *E. coli* ATCC 25922 (Table 9). These were therefore chosen for a screening on nine drug-resistant *S. aureus* strains. All the compounds retained the antibacterial activity with MIC values in the range 5.80–12.20 µM (Table 10), which are comparable to other reported FtsZ inhibitors and significantly lower than those of the reference antibiotic methicillin (MIC > 84 µM). Saturation transfer difference (STD) NMR spectroscopy is a powerful technique for the study of protein-ligand interactions in solution. This tool was utilized to characterize the binding mode of pyrimidine **51** with *Sa*FtsZ and identify its epitopes when bound to the protein. All the protons, other than the piperazine ones, showed some degree of enhancement, suggesting that **51** is bound to FtsZ. The most intense STD signal was observed at δ 7.3 ppm, which indicates that the proton of the pyrimidine is in close contact with the protein; however, the combination of the STD data with a competition STD NMR method with a known FtsZ competitive inhibitor (e.g., benzamide PC190723 or GTP) or X-ray crystallography would provide conclusive information of the actual binding site of **51** and would, therefore, fully confirm that pyrimidine **51** binds to *Sa*FtsZ. An in vitro light scattering assay showed that compound **51** inhibits the polymerization of *Sa*FtsZ activity in a concentration-dependent manner. The effect of **51** on the GTPase activity of *Sa*FtsZ revealed that it displayed moderate inhibition of the GTPase hydrolysis; however, the activity could not be measured at higher concentrations due to the poor compound solubility. Nevertheless, the authors argued that pyridine **51** suppresses FtsZ polymerization via disruption of the GTPase hydrolysis activity of the protein. Finally, the evaluation of *B. subtilis* 168 cell morphology concluded that pyridine **51** increases the cell length of the microorganism (>20 µm), compared with control cells (<5 µm).

Recently, Fang et al. reported the design, synthesis, and antibacterial evaluation of a new series of novel 2,4-disubstituted-6-thiophenyl-pyrimidine derivatives [65]. Using a similar strategy to that of Chan and co-workers, the chiral quinuclidine moiety at position 4 in compound **46** (Figure 9) was replaced by different simple and flexible cyclic or linear amine fragments. The compounds were tested on different drug-sensitive bacterial strains such as *B. subtilis* 168, *S. aureus* ATCC 29213, *E. faecium* ATCC 49624, *E. faecalis* ATCC 29212, *S. epidermidis* ATCC 12228, and *E. coli* ATCC 25922 (Table 9). It is particularly noteworthy that all the compounds displayed higher activity against the Gram-positive microorganisms and much lower MIC values on *E. coli*, likely due to the inability of the compounds to penetrate the outer membrane. The best compounds **52**, **53**, and **54** (Figure 10) of the collection were tested on a panel of drug-resistant microorganisms that included MRSA (MIC values 3.91–7.64 µM) and vancomycin-resistant *E. faecalis* and *E. faecium* (MIC values 4.02–7.82 µM) (Table 10). Remarkably, the MIC values against the latter species were 2- to 4-fold better than the reference antibiotic methicillin (MIC = 16 µM). Afterward, the authors investigated the effects of the selected compounds on the polymerization of FtsZ. By means of a light scattering assay and transmission electron microscopy, it was revealed that **54** is able to inhibit the polymerization of *Sa*FtsZ in a dose-dependent-manner. Likewise, the study of the interference of pyridine **54** with the GTPase activity of FtsZ showed that it inhibits the activity in a concentration-dependent manner. To illustrate this, compound **54** displayed an inhibition of 25% at 4 µM, a percentage that increased up to 70% at a concentration of 32 µM. The effects of **54** on the morphology of *B. subtilis* were also studied. Pleasingly, after treatment (at 4 µM), the typical microorganism shape changed from short rod cells (5–10 µm) to more elongated forms with cell lengths longer than 20 µm. A phenomenon that was also observed when this Gram-positive bacterium was treated with other FtsZ inhibitors such as benzamide and quinoline derivatives [37,42,51,53].

In a continuation of their work, the researchers identified the thiophenyl-pyrimidine **55** (Figure 10) that was able to inhibit the growth of several Gram-positive bacterial strains (including some methicillin- and vancomycin-resistant microorganisms) with MIC values ranging from 52.11 to 104.22 µM (Table 9 and Table 10), an antibacterial potency considerably higher than that of the parent quinuclidine **46** (Figure 9) [66]. The achieved results were also promising on MRSA strains, against which it displayed MIC values of 52.11 µM, 2.5-fold better than that of the reference antibiotic ampicillin (MIC = 137.50 µM). Once the antibacterial activity was assessed and confirmed, Fang et al. studied the mode of action of **55** by means of morphological studies of *B. subtilis* and *S. aureus*. Derivative **55** resulted in being able to induce cell elongation of the first species (i.e., >20 µm) and enlargement of *S. aureus*, an outcome that is once more consistent with other reported FtsZ inhibitors, such as the benzamide and the quinolinium derivatives [37,53]. STD NMR studies were undertaken in order to confirm the interaction between **55** and the protein. All the protons of pyrimidine **55** displayed a certain degree of enhancement, suggesting the direct interaction between the molecule and FtsZ. A result that is in agreement with the molecular modeling studies, in which **55** was predicted to bind into the GTP binding site and fully occupy the GDP pocket. Additionally, the docking studies showed intimate hydrophobic amide-π interactions between the A26 and A186 residues of FtsZ and the thiophenyl group.

#### 2.2.7. Quinazolines

**Figure 11:**
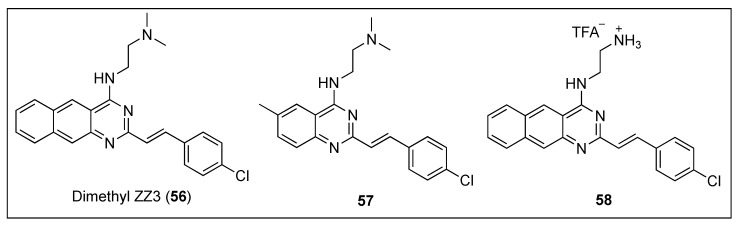
Chemical structures of dimethyl ZZ3 (**56**), quinazoline **57** and benzoquinazoline **58**.

Taking inspiration from the excellent inhibitory effects on the FtsZ GTPase activity exhibited by Zantrin Z3 (**31**) (Figure 6) and dimethyl ZZ3 (**56**) (Figure 11), Nepomuceno et al. carried out SAR studies around their benzoquinazoline cores and identified the quinazoline **57** and its analogue the benzoquinazoline **58** (Figure 11) as potent inhibitors of *Escherichia coli* FtsZ (*Ec*FtsZ) GTPase activity, with IC_50_ in the low micromolar range (Table 11) [67]. Remarkably, benzoquinazoline **58** displayed the best inhibitory activity reported to date and paved the way towards the discovery of novel potent FtsZ inhibitors bearing a quinazoline scaffold.

#### 2.2.8. Indenes, Indoles, and Benzimidazoles

**Figure 12:**
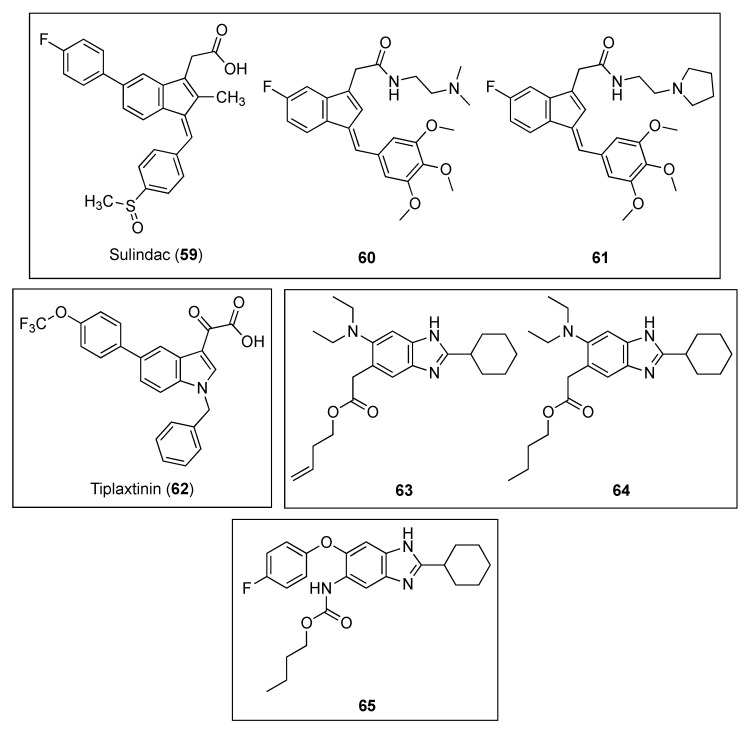
Chemical structures of the indene, indole, and benzimidazole derivatives.

Inspired by the preliminary evidence that suggests that NSAIDs (nonsteroidal anti-inflammatory drugs) possess antibacterial properties [68], Mathew and co-workers explored the in vitro anti-tubercular profile and *Mtb*FtsZ activity of a series of compounds based on the scaffold of the NSAID sulindac (**59**, Figure 12) [69]. Remarkably, indene derivatives **60** and **61** (Figure 12) exhibited notable activity against *Mtb*FtsZ with IC_50_ values of approximately 40 µM, without affecting its eukaryotic homolog tubulin at 100 µM (Table 12). Moreover, indenes **60** and **61** were able to potently inhibit the growth of *Mtb* H_37_Ra with MIC_99_ values of 72.52 and 68.49 µM, respectively, activities that correlated nicely with the observations via bright-field and fluorescence microscopy. Treatment of *Mtb* 41 with **60** and **61** led to an obvious increase in the bacterial cell length and to the absence of the distinctive mid-cell FtsZ rings. Likewise, exposure to these indene derivatives caused inhibition of the growth of *Mtb* 41. Taken all together, these results suggest that the sulindac scaffold may represent a promising chemotype for the development of novel FtsZ inhibitors against the increasingly resistant *M. tuberculosis*.

The indole oxoacetic derivative tiplaxtinin (**62**, Figure 12) is a human plasminogen activator inhibitor (PAI-1) indicated in the treatment of acute arterial thrombosis [70]. Recently, Sun and co-workers identified its promising profile as an antibacterial against Gram-positive strain and as a potent inhibitor of FtsZ [71]. Firstly, the authors studied the effects of a small library of approximately 250 compounds on *B. subtilis* morphology, which showed that tiplaxtinin (**62**) was able to cause the elongation of the rod-shaped microorganism. Evaluation of its antibacterial activity against an extended panel of clinically relevant bacterial strains concluded that tiplaxtinin is able to inhibit the growth of several Gram-positive bacteria, such as *B. subtilis*, *S. aureus*, *E. faecium*, *E. faecalis,* and *S. epidermidis* with MIC values ranging from 4.55 to 9.10 µM (Table 13). Interestingly, tiplaxtinin also exhibits potent antibacterial activity against drug-resistant strains such as MRSA and vancomycin-resistant *E. faecium* ATCC 700221. By means of a light scattering assay, the authors determined that this indole derivative enhances FtsZ polymerization in a concentration-dependent manner, a result that is in agreement with that of other FtsZ-targeting compounds already reported [52]. Finally, a 2.01 Å crystal structure of *Sa*FtsZ predicted that tiplaxtinin binds to the narrow cleft delimited by the T7-loop, the H-7 helix, and a four-stranded β-sheet.

Kumar et al. evaluated the anti-tubercular activity of two libraries of 2,5,6- and 2,5,7-trisubstituted benzimidazoles (~300 compounds) [72]. Of all the target molecules, two leads, the 2,5,6-trisubstituted benzimidazoles **63** and **64** (Figure 12) exhibited the best activities not only against the standard drug-resistant *Mtb* H37Rv strain but also against other clinical isolates of *Mtb* with MIC values in the range of 2.00–4.20 µM (Table 14). Encouragingly, none of the leads displayed appreciable cytotoxicity on Vero cells (IC_50_ > 200 µM). Afterward, the authors studied the effects of both of the compounds on the polymerization of FtsZ, a study that showed that they inhibit *Mtb*FtsZ assembly in a dose-dependent manner. Nevertheless, unexpectedly, benzimidazoles **63** and **64** provoked an enhancement in the GTPase activity, an observation that is in line with the effect that curcumin produces on *Ec*FtsZ [73]. The authors argued that the increased GTPase activity produces instability of the *Mtb*FtsZ polymer, and as a result, this leads to suppression of FtsZ polymerization and filament formation. Remarkably, scanning electron microscopy (SEM) of *Mtb* cells treated with benzimidazole **64** displayed altered bacterial cell morphology and the non-existence of septum formation, observations that are characteristic of inhibition of FtsZ assembly and cell division.

Inspired by the fact that known benzimidazoles such as albendazole and thiabendazole were found to inhibit FtsZ polymerization, Park et al. undertook the synthesis of a library of almost 400 2,5,6-trisubstituted benzimidazoles, bearing ether or thioether linkage at the 6-position [74]. Among these, benzimidazole **65** (Figure 12) exhibited the best MIC value against *Mtb* H37Rv (MIC = 1.48 µM) (Table 14). Light scattering assays, together with transmission electron microscopy, determined that benzimidazole **65** inhibits FtsZ polymerization in a dose-dependent fashion.

## 3. Conclusions and Future Outlook

A European Centre for Disease Prevention and Control (ECDC) study recently estimated that 33,000 people die each year as a result of antibacterial resistance. The report also affirms that nearly 40% of the burden is due to bacteria resistant to antibiotics of last resort (i.e., carbapenems and colistin) [75].

To make matters worse, a recent PEW Charitable Trusts analysis of the antibiotics in clinical development revealed that only eight of the candidates operate through a novel mechanism of action, and, among these, only CG400549 (**3**), brilacidin (**4**), and murepavadin (**7**) (Figure 1) are effective against the problematic multidrug-resistant ESKAPE pathogens. Consequently, as bacterial infections are becoming more arduous to treat, there is a pressing need to discover new-mechanism antibacterial agents.

This Review describes the most recent small-molecule FtsZ inhibitors that impair bacterial cytokinesis. The examples examined in this study belong to a variety of scaffolds, including benzamides, aromatic carbocycles (i.e., indenes), non-aromatic heterocycles (i.e., quinuclidines), and aromatic heterocyclic derivatives, such as quinolines, phenanthridiums, pyrimidines, quinazolines, indoles, and benzimidazoles.

The successful discovery and identification of the alkyloxybenzamide derivative PC190723 (**10**, Figure 3) as an FtsZ inhibitor incited medicinal chemistry efforts towards the exploration of the FtsZ protein as a novel antibacterial target. Its potent and selective anti-staphylococcal activity, with MIC values as low as 2.81 µM, validated FtsZ as an antibacterial target and served as a promising starting point for the development of other FtsZ-targeting inhibitors. Bearing the same chemical scaffold, benzamide TXA707 (**12**, Figure 3), with improved drug-like properties and enhanced anti-staphylococcal activity compared to PC190723, is the most advanced FtsZ-targeting antibacterial agent. Even if TXA707 does eventually not progress to the market, the acquired structural data can be used to design and develop new inhibitors that target FtsZ. Additionally, it has recently been demonstrated that TXA707 acts synergistically with the third-generation cephalosporin cefdinir against several multidrug-resistant *S. aureus* [76]. Combination therapies are successfully being used to enhance drug efficacy and/or broaden drug spectrum in anticancer, HIV, and anti-tubercular therapies, and they could perhaps be implemented in the FtsZ field to gain lower MIC values to overcome any potential resistance issues or to expand the spectrum of activity to include Gram-negative microorganisms. In this regard, investigations on the combination of PC190723 prodrug (TXY436) with an efflux-pump inhibitor (i.e., PAβN) revealed that the presence of sub-inhibitory concentrations of PAβN confers TXY436 with activity against Gram-negative strains (namely, *E. coli*, *P. aeruginosa,* and *A. baumannii*) [77].

It is hoped that these fruitful stories, combined with the earlier reported FtsZ inhibitors, will lay the foundation for the development of a new class of antibacterial chemical entities with significant therapeutic value that operate through inhibition of bacterial cytokinesis.

## Figures and Tables

**Table 1:** Minimum inhibitory concentrations of the benzamide analogues.

Microorganisms	MIC Values (μM)							
	PC190723 (10)	15	16	18	19	20	21	22
*B. subtilis* 168	2.81	−	−	−	−	−	−	−
*B. subtilis* ATCC 9372	−	2.97	0.88	−	0.04	>154.42	0.65	0.29
*B. cereus* ATCC 14579	2.81	−	−	−	−	−	−	−
*S. pumilus* CMCC 63202	−	−	−	−	0.08	0.14	5.24	2.35
*S. aureus* ATCC 25923	−	2.97	3.50	−	5.18	2.41	2.62	2.35
*S. aureus* ATCC 29213	2.81	5.95	28.04	3.35	−	−	−	−
*S. aureus* ATCC 43300 *	2.81	−	−	−	5.18	4.83	5.24	2.35
*S. aureus* ATCC BAA-44 **	2.81	−	−	−	−	−	−	−
*S. aureus* PR ***	−	5.95	28.04	−	10.35	4.83	5.24	2.35
*S. epidermidis* ATCC 12228	2.81	−	−	−	−	−	−	−
*S. haemolyticus* ATCC 29970	1.41	−	−	−	−	−	−	−
*S. hominis* ATCC 27844	2.81	−	−	−	−	−	−	−
*S. lugdunensis* ATCC 43809	2.81	−	−	−	−	−	−	−
*S. saprophyticus* ATCC 15305	2.81	−	−	−	−	−	−	−
*S. warneri* ATCC 49454	2.81	−	−	−	−	−	−	−
*S. pyogenes* PS ****	−	190.37	>448.60	−	−	−	−	−
*S. pyogenes* ATCC 51339	>179.91	−	−	−	−	−	−	−
*S. pneumoniae* ATCC 49619	>179.91	>380.75	224.30	−	−	−	−	−
*E. coli* ATCC 25922	>179.91	380.75	897.21	>214.5	>165.63	>154.42	>167.66	>150.17
*H. influenzae* ATCC 49247	>179.91	−	−	−	−	−	−	−
*P. aeruginosa* ATCC 27853	>179.91	380.75	448.60	−	>165.63	>154.42	>167.66	>150.17

* MRSA; ** MDRSA; *** S. aureus PR: penicillin-resistant strain isolated clinically, not characterized; **** *S. pyogenes* PS: penicillin-susceptible strain.

**Table 2:** Combination studies of benzamide **18** with diverse β-lactam antibiotics.

MRSA Strain Numbers	MIC Values (µM)										
	18	ME	18 + ME	CL	18 + CL	AM	18 + AM	CX	18 + CX	MR	18 + MR
417 *	107.5	2691.76	5.26	146.83	4.59	1401.20	21.89	2412.90	4.71	166.90	10.43
2516 *	127.5	168.24	10.51	36.71	4.59	175.15	21.89	1206.45	4.71	41.72	10.43
774 *	1716	42.06	10.51	4.59	2.29	1401.20	21.89	2412.90	4.71	83.45	10.43

* Clinically isolated MRSA strain.

**Table 3:** Minimum inhibitory concentrations of the quinolinium and structurally related compounds.

Microorganisms	MIC Values (µM)									
	24	26	27	28	29	30	33	34	35	36
*B. subtilis* 168	7.01	8.29	4.16	2.72	2.72	0.38	2.81	8.07	8.07	3.61
*S. aureus* ATCC 29213	3.50	8.29	4.16	2.72	2.72	0.38	2.81	2.02	2.02	1.81
*S. aureus* ATCC BAA-41 *	3.50	8.29	4.16	5.45	2.72	1.54	2.81	4.04	4.04	1.81
*S. aureus* ATCC 33591 *	−	−	−	5.45	2.72	1.54	−	−	−	−
*S. aureus* ATCC 43300 *	−	−	−	2.72	2.72	1.54	−	4.04	8.07	3.61
*S. aureus* ATCC 33592 *	−	−	−	−	−	0.77	−	−	−	−
*E. faecium* ATCC 49624	7.01	12.44	8.31	1.36	3.62	−	3.74	4.04	4.04	1.81
*E. faecium* ATCC 700221 **	7.01	12.44	8.31	2.72	3.62	−	3.74	8.07	8.07	3.61
*E. faecalis* ATCC 29212	7.01	−	−	1.36	3.62	6.16	2.81	8.07	8.07	3.61
*E. faecalis* ATCC 700221	−	−	−	−	−	1.54	−	−	−	−
*E. faecalis* ATCC 51575 **	−	−	−	−	3.62	−	2.81	−	−	−
*S. epidermidis* ATCC 12228	3.50	−	−	1.36	1.81	−	1.40	−	−	−
*S. pyogenes* ATCC 12344	−	−	−	2.72	−	−	−	−	−	−
*E. coli* ATCC 25922	56.06	16.59	12.47	5.45	5.43	1.54	5.61	32.30	16.15	14.45
*E. coli* ATCC BAA-2469 ***	−	16.59	12.47	5.45	5.43	1.54	5.61	32.30	32.30	28.91
*P. aeruginosa* ATCC BAA-2108 ***	−	132.69	99.72	10.90	10.86	6.16	11.23	>129.19	>129.19	115.64
*K. pneumoniae* ATCC BAA−2470	−	132.69	99.72	−	−	12.31	−	>129.19	>129.19	115.64
*K. pneumoniae* ATCC BAA-1144	112.11	−	−	87.20	−	−	44.91	−	−	−
*A. baumannii* ATCC 19606	−	−	−	87.20	−	12.31	−	64.59	32.30	14.45

* MRSA strain; ** vancomycin-resistant strain; *** multidrug-resistant strain.

**Table 4:** MIC values of β-lactam antibiotics in combination with quinolinium **33**.

Drug-Resistant *S. Aureus* Strains	β-Lactam Antibiotics	MIC Values Single Antibiotic (µM)	MIC Values Combination of β-Lactam Antibiotic with 33 (µM)
ATCC 29247 *	Ampicillin	68.69	17.17
ATCC BAA-41 **	Ampicillin	137.38	34.34
ATCC BAA-41 **	Methicillin	2691.76	84.11
ATCC BAA-41 **	Oxacillin	637.71	79.71
ATCC BAA-41 **	Imipenem	80.17	20.04
ATCC BAA-41 **	Ceftazidime	175.64	43.91

* ampicillin-resistant *S. aureus* strain; ** MRSA strain.

**Table 5:** Biological evaluation of 1,2-dihydroquinolines analogues **38** and **39**.

Compounds	*M. Smegmatis* MIC Values (µM)	*Mtb*FtsZ IC_50_ Values (µM)	
		Light Scattering Assay	GTPase Assay
**38**	2.58	77.30	78.50
**39**	22.30	33.40	39.00

**Table 6:** MIC values of the phenanthridium analogues.

Microorganisms	MIC Values (µM)					
	40	41	42	43	44	45
*B. subtilis* ATCC 9372	14.71	12.75	15.52	0.15	0.32	0.31
*S. pumilus* ATCC 63202	−	−	−	0.15	0.32	0.31
*S. aureus* ATCC 25923	29.43	51	248.29	0.15	0.15	0.15
*S. aureus* ATCC 29213 *	235.48	204.01	124.15			
*S. aureus* ATCC 43300 *	−	−	−	0.64	1.26	0.62
*S. aureus* PR **	58.87	25.50	15.52	0.64	0.63	2.48
*S. epidermidis* **	235.48	102.01	496.59	0.32	0.32	0.62
*S. pyogenes* PS ***	14.71	12.75	15.52	2.58	2.53	4.95
*S. pyogenes* PR **	29.43	25.50	31.04	5.16	5.05	9.91
*E. coli* ATCC 25922	>470.95	>408.03	>496.59	>165.02	>161.64	>158.48
*P. aeruginosa* ATCC 27853	>470.95	>408.03	>496.59	>165.02	>161.64	>158.48

* MRSA strain; ** penicillin-resistant strain isolated clinically, not characterized; *** penicillin-susceptible strain.

**Table 7:** Minimum inhibitory concentrations of the quinuclidine analogues.

Microorganisms	MIC Values (µM)		
	46	47	48
*B. subtilis* 168	-	-	50.50
*S. aureus* ATCC 29213	897	24.60	50.50
*S. aureus* ATCC 29247 *	-	-	50.50
*S. aureus* ATCC BAA-41 **	-	-	50.50
*S. aureus* ATCC BAA-44 ***	-	-	50.50
*E. faecium* ATCC 49624	-	-	50.50
*E. faecium* ATCC 700221 ****	-	-	50.50
*E. faecalis* ATCC 29212	-	-	50.50
*E. coli* ATCC 25922	449	49.20	75.50

* Ampicillin-resistant strain; ** MRSA strain; *** multidrug-resistant strain; **** vancomycin-resistant strain.

**Table 8:** Combination studies of quinuclidine **48** with diverse β-lactam antibiotics.

Drug-Resistant *S. Aureus* Strains	MIC Values (µM)											
	AP	48 + AP	OX	48 + OX	ME	48 + ME	IM	48 + IM	CF	48 + CF	CZ	48 + CZ
**ATCC 29247** *	68.76	17.17	−	−	−	−	−	−	−	−	−	−
**ATCC BAA-41** **	103.03	25.76	637.71	79.71	2691.76	672.94	53.45	13.36	598.89	74.86	58.55	7.32

* ampicillin-resistant *S. aureus* strain; ** MRSA strain.

**Table 9:** MIC values of the pyrimidine analogues on drug-sensitive strains.

Microorganisms	MIC Values (µM)						
	49	50	51	52	53	54	55
*B. subtilis* 168	−	−	−	3.91	7.64	4.02	52.11
*S. aureus* ATCC 29213	5.80	6.10	7.82	3.91	7.64	4.02	52.11
*E. faecium* ATCC 49624	−	−	−	7.82	7.64	4.02	104.22
*E. faecalis* ATCC 29212	−	−	−	3.91	7.64	4.02	104.22
*S. epidermidis* ATCC 12228	−	−	−	3.91	7.64	4.02	52.11
*E. coli* ATCC 25922	>123.65	>130.17	>125.08	250.13	244.45	257.18	208.44

**Table 10:** MIC values of the pyrimidine analogues on drug-resistant strains.

Microorganisms	MIC Values (µM)						
	49	50	51	52	53	54	55
*S. aureus* ATCC 29247 *	5.80	6.10	7.82	−	−	−	52.11
*S. aureus* ATCC BAA−1717 **	5.80	6.10	7.82	−	−	−	52.11
*S. aureus* BAA−1720 **	11.59	12.20	11.73	3.91	7.64	4.02	52.11
*S. aureus* BAA−41 **	11.59	12.20	11.73	3.91	7.64	4.02	52.11
*S. aureus* ATCC 43300 **	11.59	12.20	11.73	3.91	7.64	4.02	−
*S. aureus* USA300 #417 **	11.59	12.20	11.73	−	−	−	−
*S. aureus* USA300 #757 **	11.59	12.20	11.73	−	−	−	−
*S. aureus* USA300 #1799 **	11.59	12.20	11.73	−	−	−	−
*S. aureus* USA300 #2690 **	11.59	12.20	11.73	−	−	−	−
*S. aureus* ATCC 33591 **	−	−	−	3.91	7.64	4.02	−
*S. aureus* ATCC 33592 **	−	−	−	3.91	7.64	4.02	−
*E. faecalis* ATCC 51575 ***	−	−	−	7.82	7.64	4.02	104.22
*E. faecium* ATCC 700221 ***	−	−	−	7.82	7.64	4.02	104.22
*E. coli* ATCC BAA−2469 ****	−	−	−	−	−	−	208.44
*P. aeruginosa* ATCC BAA−2108 ****	−	−	−	−	−	−	>208.44
*K. pneumoniae* ATCC BAA−1144 ****	−	−	−	−	−	−	>208.44

* Ampicillin-resistant strain; ** MRSA strain; *** vancomycin-resistant strain; **** multidrug-resistant strain.

**Table 11:** IC_50_ values of quinazolines and related compounds.

Compound	*Ec*FtsZ IC_50_ (µM)
Zantrin Z3 (**31**)	24.00
Dimethyl ZZ3 (**56**)	12.00
**57**	24.00
**58**	9.00

**Table 12:** Biological evaluation of indenes **60** and **61**.

Compounds	*Mtb* FtsZ IC_50_ (µM)	Tubulin Polymerization IC_50_ (µM)	*Mtb* H_37_Rv IC_90_ (µM)	*Mtb* H_37_Ra MIC_99_ (µM)	MAC NJ211 MIC_99_ (µM)
**60**	43.30	NA	>100	72.52	>145.28
**61**	37.60	NA	25.48	68.49	>143.32

NA = not active up to 100 µM.

**Table 13:** MIC values of tiplaxtinin (**62**) on Gram-positive and Gram-negative bacteria.

Microorganisms	MIC Values (µM)
	62
*B. subtilis* 168	4.55
*S. aureus* ATCC 29213	4.55
*S. aureus* ATCC 29247 *	4.55
*S. aureus* ATCC 33591 **	9.10
*S. aureus* ATCC 43300 **	9.10
*S. aureus* ATCC BAA-41 **	9.10
*S. aureus* ATCC BAA-1717 **	9.10
*S. aureus* ATCC BAA-1720 **	9.10
*S. aureus* ATCC BAA-1747 **	9.10
*S. aureus* USA300 #417 **	4.55
*S. aureus* USA300 #2690 **	4.55
*E. faecium* ATCC 49624	9.10
*E. faecium* ATCC 700221 ***	9.10
*E. faecalis* ATCC 29212	9.10
*S. epidermidis* ATCC 12228	4.55
*E. coli* ATCC 25922	>109.24
*P. aeruginosa* ATCC BAA-2108 ****	>109.24
*K. pneumoniae* ATCC BAA-1144 ****	>109.24

* Ampicillin-resistant strain; ** MRSA strain; *** vancomycin-resistant strain; **** multidrug-resistant strain.

**Table 14:** Antimicrobial activities of benzimidazoles **63, 64**, and **65** against *M. tuberculosis* strains.

Compounds	MIC_99_ *Mtb* Strains (µM)						IC_50_ Vero Cells (µM)
	H37Rv	W210	NHN20	NHN335	NHN382	TN587	
**63**	4.20	4.20	4.20	4.20	4.20	4.20	>400
**64**	4.00	4.00	4.00	4.00	2.00	4.00	>400
**65**	1.48	-	-	-	-	-	60
